# Assessment of Receipt of the First Home Health Care Visit After Hospital Discharge Among Older Adults

**DOI:** 10.1001/jamanetworkopen.2020.15470

**Published:** 2020-09-02

**Authors:** Jun Li, Mingyu Qi, Rachel M. Werner

**Affiliations:** 1 Department of Health Management and Policy, School of Public Health, University of Michigan, Ann Arbor; 2 Now with Department of Public Administration and International Affairs, The Maxwell School of Citizenship and Public Affairs, Syracuse University, Syracuse, New York; 3 Department of Medicine, Perelman School of Medicine, University of Pennsylvania, Philadelphia; 4 Leonard Davis Institute of Health Economics, University of Pennsylvania, Philadelphia; 5 Center for Health Equity Research and Promotion, Corporal Michael J. Crescenz VA Medical Center, Philadelphia, Pennsylvania

## Abstract

**Importance:**

Home health care is one of the fastest growing postacute services in the US and is increasingly important in the era of coronavirus disease 2019 and payment reform, yet it is unknown whether patients who need home health care are receiving it.

**Objective:**

To examine how often patients referred to home health care at hospital discharge receive it and whether there is evidence of disparities.

**Design, Setting, and Participants:**

This cross-sectional study used Medicare data regarding the postacute home health care setting from October 1, 2015, through September 30, 2016. The participants were Medicare fee-for-service and Medicare Advantage beneficiaries who were discharged alive from a hospital with a referral to home health care (2 379 506 discharges). Statistical analysis was performed from July 2019 to June 2020.

**Exposures:**

Hospital referral to home health care.

**Main Outcomes and Measures:**

Primary outcomes included whether discharges received their first home health care visit within 14 days of hospital discharge and the number of days between hospital discharge and the first home health visit. Differences in the likelihood of receiving home health care across patient, zip code, and hospital characteristics were also examined.

**Results:**

Among 2 379 506 discharges from the hospital with a home health care referral, 1 358 697 patients (57.1%) were female, 468 762 (19.7%) were non-White, and 466 383 (19.6%) were dually enrolled in Medicare and Medicaid; patients had a mean (SD) age of 73.9 (11.9) years and 4.1 (2.1) Elixhauser comorbidities. Only 1 284 300 patients (54.0%) discharged from the hospital with a home health referral received home health care services within 14 days of discharge. Of the remaining 1 095 206 patients (46.0%) discharged, 37.7% (896 660 discharges) never received any home health care, while 8.3% (198 546 discharges) were institutionalized or died within 14 days without a preceding home health care visit. Patients who were Black or Hispanic received home health at lower rates than did patients who were White (48.0% [95% CI, 47.8%-48.1%] of Black and 46.1% [95% CI, 45.7%-46.5%] of Hispanic discharges received home health within 14 days compared with 55.3% [95% CI, 55.2%-55.4%] of White discharges). In addition, disadvantaged patients waited longer for their first home health care visit. For example, patients living in high-unemployment zip codes waited a mean of 2.0 days (95% CI, 2.0-2.0 days), whereas those living in low-unemployment zip codes waited 1.8 days (95% CI, 1.8-1.8 days).

**Conclusions and Relevance:**

Disparities in the use of home health care remain an issue in the US. As home health care is increasingly presented as a safer alternative to institutional postacute care during coronavirus disease 2019, and payment reforms continue to pressure hospitals to discharge patients home, ensuring the availability of safe and equitable care will be crucial to maintaining high-quality care.

## Key Points

**Question:**

How often do Medicare patients referred to home health care at hospital discharge receive a home health care visit, and are there disparities?

**Findings:**

In this cross-sectional study of Medicare beneficiaries in 2016, only 54.0% of patients discharged from the hospital with a home health care referral received home health care services within 14 days of discharge. This rate was even lower among Black and Hispanic patients, those who were dually enrolled in both Medicare and Medicaid, and patients who lived in high-poverty, high-unemployment zip codes.

**Meaning:**

These findings suggest that patients may face important differential barriers in access to home health care.

## Teaser

This cross-sectional study examines Medicare patients who were discharged from the hospital with a home health care referral in 2016 and the rates at which home health care was actually received.

## Introduction

The use of postacute care has grown substantially over the past several decades.^1^ More than 40% of Medicare beneficiaries receive postacute care after a hospital discharge, and most of those beneficiaries received care from a home health agency.^2^ Home health care is one of the most rapidly growing postacute services used by Medicare patients after discharge from hospitals.^1,2,3^

Despite the importance of home health care delivery, it is unknown how often patients who need home health care actually get it. A prior study^4^ found that between 2006 and 2011, approximately 30% of Medicare beneficiaries referred to home health care after hospital discharge did not receive it. Since 2011, the implementation of large-scale payment reforms has been associated with increased use of and need for home health care.^5,6,7^ As patients are increasingly being discharged home rather than to institutional postacute care settings, and hospitals are being held accountable for readmissions, home health care may be critical to successfully and safely transitioning patients out of the hospital.

Our objective was therefore to describe how often home health referrals were successful—that is, how often home health agencies provided at least 1 home visit after a discharge from the hospital with a home health referral—using recent national data. We further examined whether the rate of successful home health referral varied by patient sociodemographic characteristics, hypothesizing that socioeconomically disadvantaged patients may be less likely to receive home health care when referred.

## Methods

The University of Pennsylvania institutional review board approved the study and waived the Health Insurance Portability and Accountability Act informed consent because this study involved secondary analysis of existing data. This study followed the Strengthening the Reporting of Observational Studies in Epidemiology (STROBE) reporting guideline.

### Study Data and Study Population

We used 100% Medicare Provider Analysis Review (MedPAR) claims data, including Medicare fee-for-service and Medicare Advantage beneficiaries who were discharged alive from a hospital with a referral to home health care in fiscal year 2016, between October 1, 2015, and September 30, 2016. Patients were considered referred to home health care if they had a discharge destination of home health in the MedPAR data. Patients were included if they were discharged from an acute care or critical access hospital with a discharge destination of home health and were continuously enrolled in either fee-for-service Medicare or Medicare Advantage from hospital admission through 60 days after discharge. Although not all hospital claims data for Medicare Advantage patients are captured in MedPAR data, prior literature^8^ indicates that hospitals that report Medicare Advantage data account for 92% of all US hospital discharges. Thus, missing Medicare Advantage information from the small subset of hospitals has minimal impact on our findings.

To evaluate the reliability of referral status, we assessed whether the discharge destination information captured by MedPAR was accurate for patients referred to skilled nursing facility care following hospital discharge. Like home health care, skilled nursing facility care is one of the most common types of postacute care for Medicare patients. We found that 93.2% of patients documented as referred to skilled nursing facilities received care within 14 days, whereas the remaining 6.8% of patients may not have received the intended care or were inaccurately coded. This suggests that the MedPAR discharge destination field is generally an accurate reflection of where patients are intended to go after hospital discharge.

We used several data sources. We used the Outcome and Assessment Information Set, containing home health assessments for all Medicare beneficiaries, to identify discharges receiving home health care within 14 days of hospital discharge. We used the Minimum Data Set and the Inpatient Rehabilitation Facility Patient Assessment Instrument, containing nursing home and inpatient rehabilitation assessments, respectively, to identify discharges to institutional postacute care within 14 days of hospital discharge. We used the MedPAR to identify rehospitalizations within 14 days of hospital discharge. We used the Medicare Beneficiary Summary File to determine whether patients who were discharged died within 14 days of hospital discharge. We supplemented these data with the American Community Survey 2012 to 2016 file to measure zip code–level socioeconomic characteristics, the Medicare Provider of Service file to measure most hospital characteristics, and Medicare cost reports to identify whether a hospital owned (or was vertically integrated with) a home health agency.

### Outcome Variables: Home Health Utilization

Our main objective was to examine whether patients discharged from the hospital with the intention of receiving home health (as indicated in the hospital discharge data, MedPAR) actually received home health care. We measured this in 2 ways. First, we determined whether discharged patients had their first home health visit within 14 days of hospital discharge. Although more than 80% of initial home health visits after hospital discharge occur within 2 days of discharge, Medicare fee-for-service covers postdischarge home health visits under Part A if they occur within 14 days of hospital discharge.^9^ We thus used 14 days as our cutoff for receipt of postdischarge home health. Second, we measured the number of days between hospital discharge and the first home health visit. Timely care is a measure used by Medicare to evaluate home health quality,^10^ consistent with prior literature suggesting an association between better outcomes and shorter wait times.^11^ Additionally, Medicare requires a home health agency to conduct an initial visit within 48 hours of a referral, of the patient’s return home, or on the physician-ordered start of care date.^12^ Therefore, we examine the wait times patients face to determine whether disparities exist along this dimension.

We further classified discharges into 2 additional groups: (1) those who did not receive any home health care and survived at least 14 days after discharge from the hospital and (2) those who received institutional postacute care, were rehospitalized, or died prior to receiving home health care within 14 days of hospital discharge. We considered a discharge to have successfully received home health only if the first home health visit occurred prior to any institutional postacute care or rehospitalization.

### Explanatory Variables: Patient, Zip Code, and Hospital Characteristics

We examined differences in the likelihood of receiving home health care across patient, zip code, and hospital characteristics. Patient characteristics included age, sex, race/ethnicity, enrollment in Medicare fee-for-service vs Medicare Advantage, dual enrollment in Medicare and Medicaid, comorbidity burden (using Elixhauser comorbidities), and residence in an urban vs nonurban zip code (measured using Rural-Urban Commuting Area Codes indicating metropolitan area or not).

We also examined 2 socioeconomic characteristics of a patient’s zip code of residence—living in a zip code with a high unemployment rate (the top quartile of unemployment rates by zip code in each state) or not and living in a zip code with a high poverty rate (the top quartile of the percentage living in poverty by zip code in each state) or not. Finally, we examined characteristics of the discharging hospital, including profit status, teaching status, urban vs rural location, large size (≥250 beds), and whether it had a vertically integrated home health agency.

### Statistical Analysis

We calculated the proportions of patients that received a home health visit and the mean time to the first home health visit using SAS version 9.4 (SAS Institute). Statistical analysis was performed from July 2019 to June 2020.

## Results

**Figure 1.**
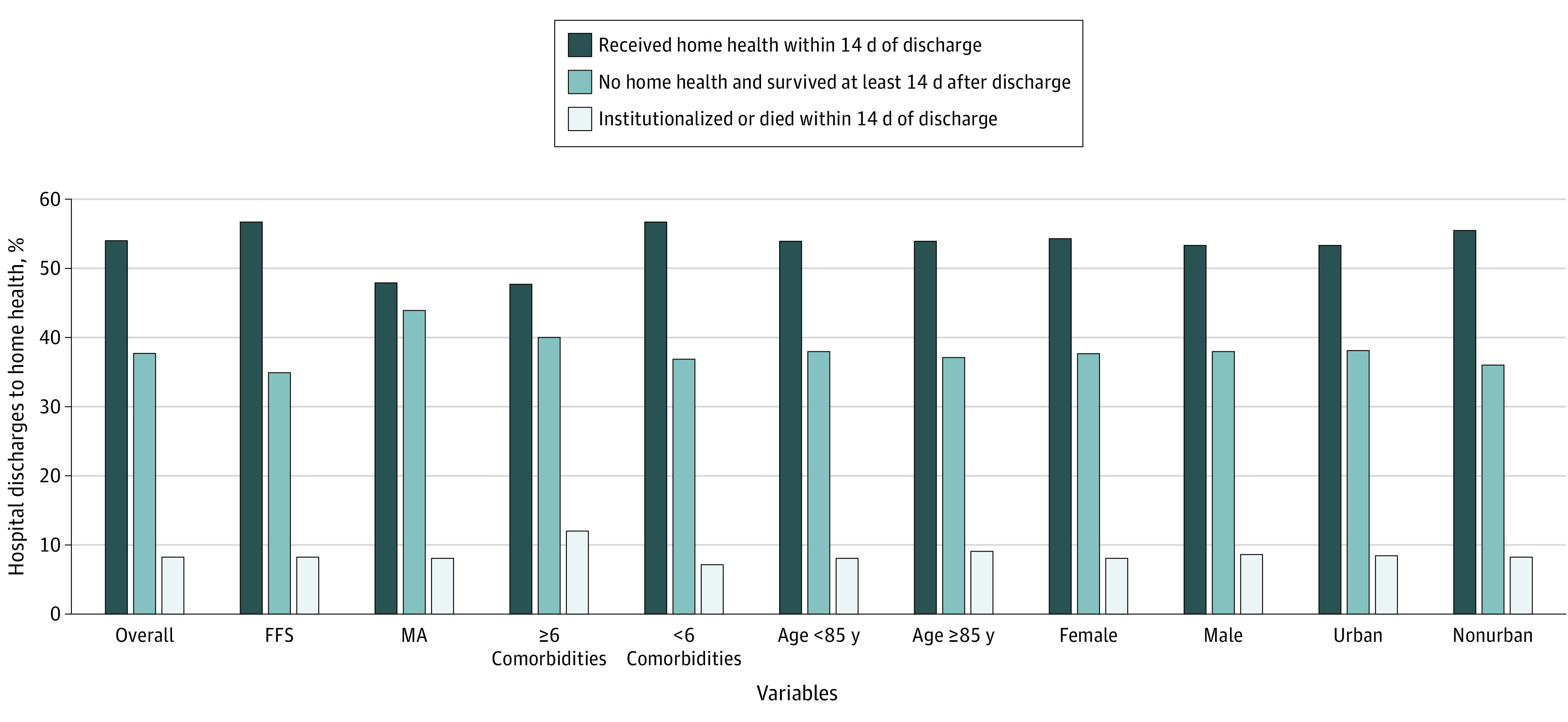
Postdischarge Status by Patient Characteristics Among Medicare Patients Referred to Home Health Care FFS indicates fee-for-service; MA, Medicare Advantage.

Among the 2 379 506 patients discharged from the hospital with a home health care referral, 1 358 697 (57.1%) were female, 468 762 (19.7%) were non-White, and 466 383 (19.6%) were dually enrolled in Medicare and Medicaid; patients had a mean (SD) age of 73.9 (11.9) years and 4.1 (2.1) Elixhauser comorbidities. Of these, only 1 284 300 (54.0%) received at least 1 home health visit within 14 days of discharge (Figure 1). Of the remaining 1 095 206 patients (46.0%), more than one-third of discharges (896 660 discharges [37.7%]) never received any home health care following hospital discharge despite being referred to home health care, while 8.3% (198 546 discharges) were institutionalized or died within 14 days without a preceding home health visit. Among them, 6.0% (144 731 discharges) were rehospitalized, 1.6% (38 211 discharges) were admitted to institutional postacute care, and 0.7% (15 604 discharges) died within 14 days. Initial home health visit rates were higher among fee-for-service Medicare beneficiaries compared with those enrolled in Medicare Advantage (56.7% [95% CI, 56.6%-56.8%] vs 47.9% [95% CI, 47.8%-48.0%]) but were lower among discharges with 6 or more comorbidities (47.7% [95% CI, 47.6%-47.9%] vs 56.1% [95% CI, 56.0%-56.2%]), a difference that was partially explained by higher mortality and institutionalization rates among these discharges. Rates of home health visits were similar by age, sex, and urban residence.

**Figure 2.**
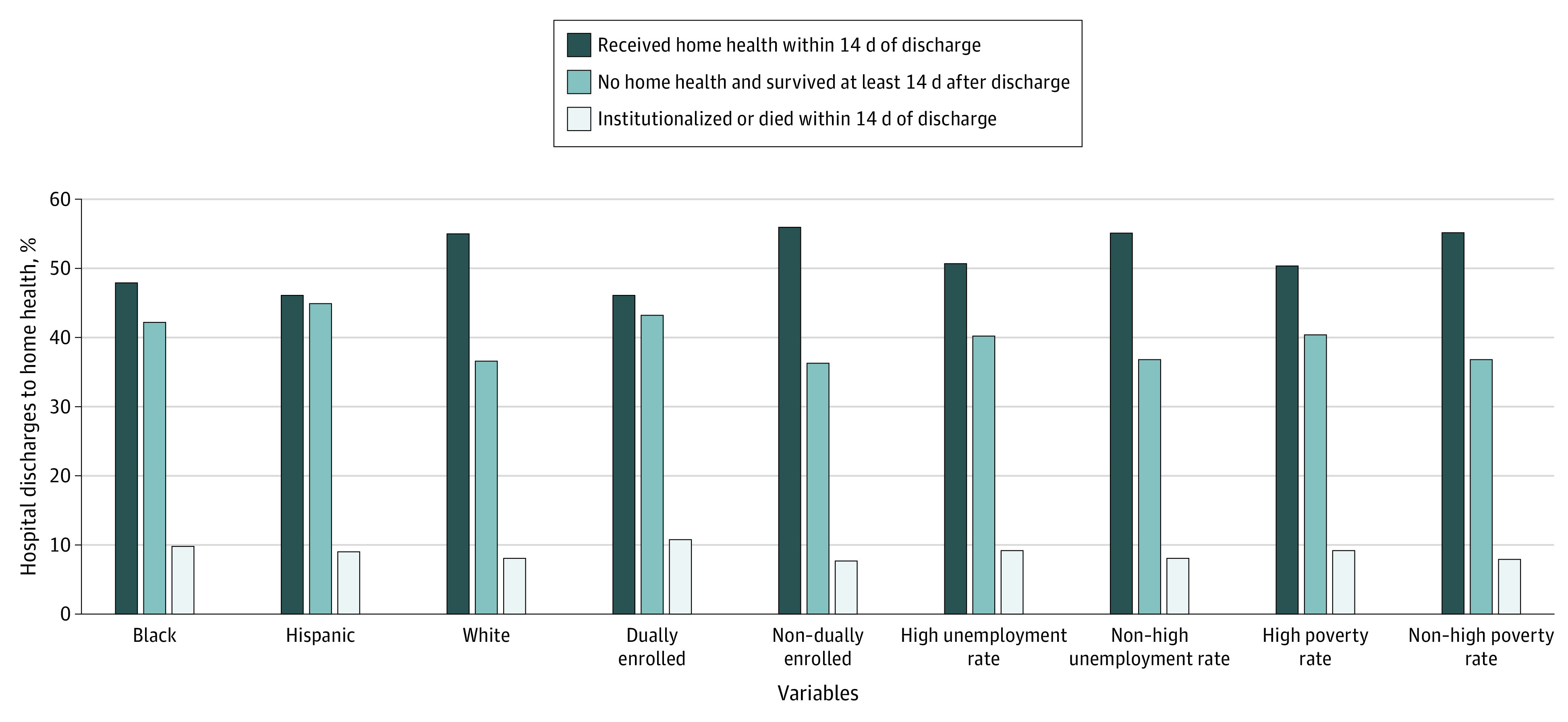
Postdischarge Status by Race/Ethnicity and Socioeconomic Characteristics Among Medicare Patients Referred to Home Health Care

Socioeconomically disadvantaged patients were less likely to receive home health care when referred at hospital discharge (Figure 2). Patients who were Black or Hispanic received home health at lower rates than did patients who were White (48.0% [95% CI, 47.8%-48.1%] of Black and 46.1% [95% CI, 45.7%-46.5%] of Hispanic discharges received home health within 14 days compared with 55.3% [95% CI, 55.2%-55.4%] of White discharges). Patients who were dually enrolled in Medicare and Medicaid received home health care at lower rates than did patients who were not (46.1% [95% CI, 45.9%-46.2%] vs 55.9% [95% CI, 55.9%-56.0%]). This pattern also held for patients living in zip codes with markers of lower vs higher socioeconomic status, with lower home health visits in zip codes with high unemployment rates (50.7% [95% CI, 50.5%-50.8%] vs 55.1% [95% CI, 55.0%-55.2%]) and high poverty rates (50.4% [95% CI, 50.3%-50.5%] vs 55.2% [95% CI, 55.1%-55.3%]).

**Figure 3.**
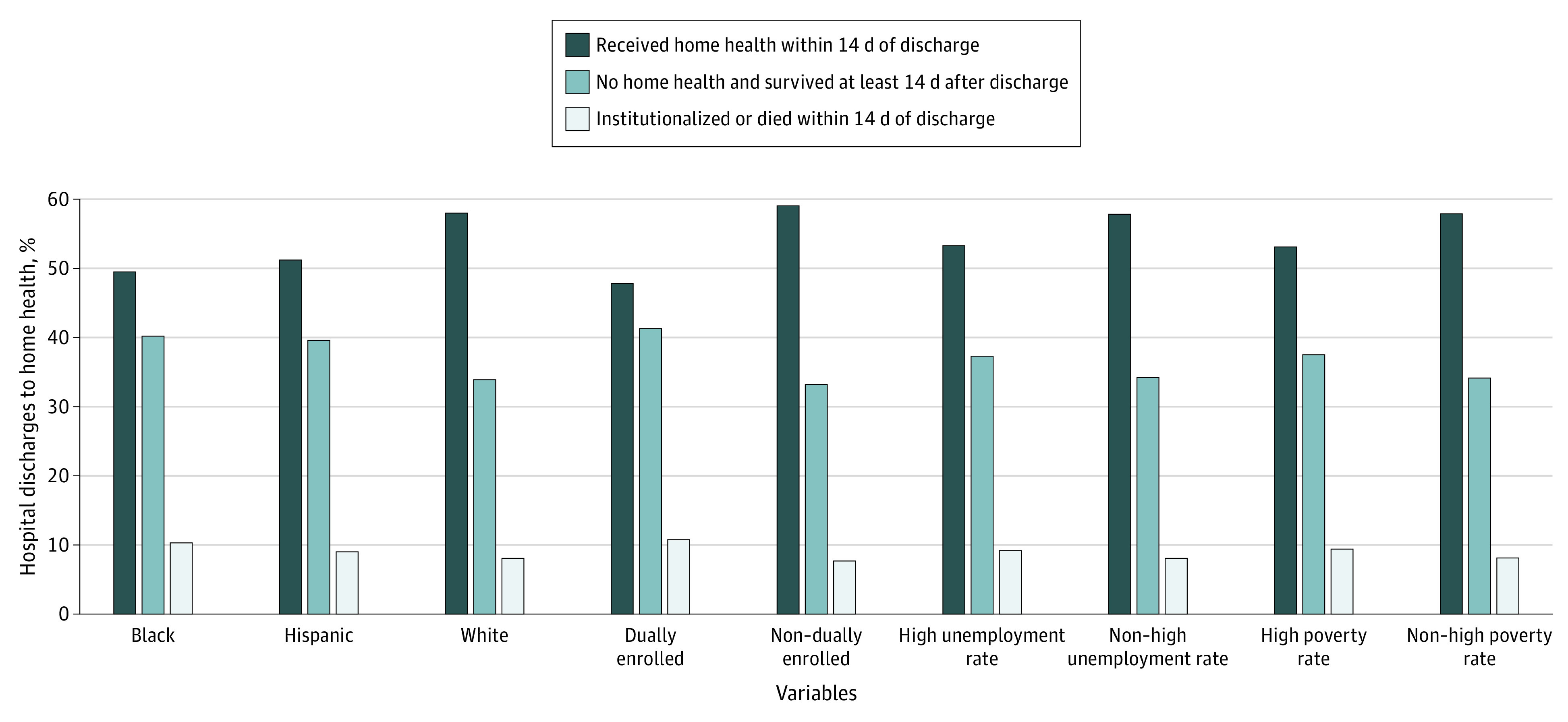
Postdischarge Status by Race/Ethnicity and Socioeconomic Characteristics Among Fee-for-Service Medicare Beneficiaries Referred to Home Health Care

To test whether differences in rates of first home health visits among socioeconomically disadvantaged patients was associated with differences in enrollment in Medicare Advantage, which may actively manage home health utilization and only approve its use in certain circumstances, we repeated these summaries among 1 643 830 fee-for-service-enrolled discharges. The lower rates of home health visits among socioeconomically disadvantaged patients remained, as shown in Figure 3.

**Figure 4.**
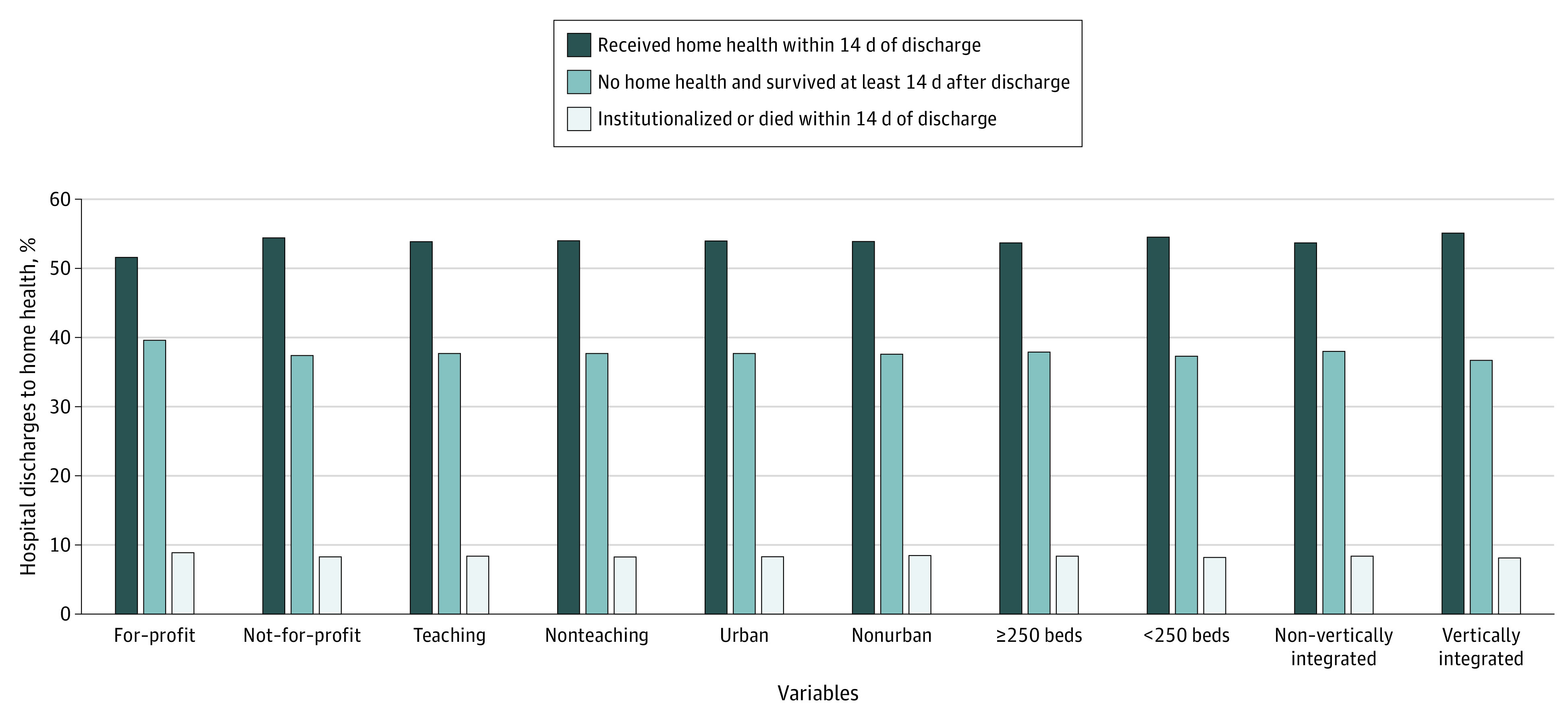
Postdischarge Status by Hospital Characteristics Among Medicare Patients Referred to Home Health Care

The differences in first home health visits by hospital characteristic were smaller (Figure 4). Discharges with a home health referral from for-profit hospitals had slightly lower rates of home health visits compared with discharges from not-for-profit hospitals (51.6% [95% CI, 51.4%-51.7%] vs 54.4% [95% CI, 54.3%-54.4%]) as did discharges from hospitals that were not vertically integrated with a home health agency compared with hospitals that were (53.7% [95% CI, 53.6%-53.7%] vs 55.1% [95% CI, 54.9%-55.2%]). There were no meaningful differences in receipt of home health care by teaching status, urban location, or hospital size.

Socioeconomically disadvantaged patients also waited longer, on average, before receiving their first home health visit (Table). Across all patients, the mean number of days between hospital discharge and the first home health visit was 1.8 days (95% CI, 1.8-1.8 days). There were longer intervals between discharge and the first home health visit for discharges among Black (2.2 days [95% CI, 2.2-2.2 days]) and Hispanic (2.1 days [95% CI, 2.0-2.1 days]) patients compared with White patients (1.8 days [95% CI, 1.8-1.8 days]), dually enrolled patients compared with non–dually enrolled patients (2.1 days [95% CI, 2.1-2.1 days] vs 1.8 days [95% CI, 1.8-1.8 days]), and patients living in high-unemployment and high-poverty zip codes compared with their low-unemployment (2.0 days [95% CI, 2.0-2.0 days] vs 1.8 days [95% CI, 1.8-1.8 days]) and low-poverty (2.0 days [95% CI, 2.0-2.0 days] vs 1.8 days [95% CI, 1.8-1.8 days]) counterparts.

**Table.** Time Between Hospital Discharge and First Home Health Care Visit Among Patients Referred to Home Health Care^a^

Characteristic	Time between hospital discharge and first home health care visit, mean (95% CI), d (N = 1 284 300 patients)
Race/ethnicity	
White	1.8 (1.8-1.8)
Black	2.2 (2.2-2.2)
Hispanic	2.1 (2.0-2.1)
Medicare status	
Enrolled in Medicare Fee-for-Service	1.8 (1.8-1.8)
Enrolled in Medicare Advantage	2.0 (2.0-2.0)
Dually enrolled in Medicare and Medicaid	2.1 (2.1-2.1)
Non–dually enrolled in Medicare and Medicaid	1.8 (1.8-1.8)
Elixhauser comorbidities	
<6	1.8 (1.8-1.8)
≥6 (top quartile)	2.1 (2.1-2.1)
Characteristics of patient's residential zip code	
High unemployment rate (top quartile)^b^	2.0 (2.0-2.0)
Non–high unemployment rate^b^	1.8 (1.8-1.8)
High poverty rate (top quartile)	2.0 (2.0-2.0)
Non–high poverty rate^b^	1.8 (1.8-1.8)

^a^ Calculated using Medicare data from October 2015 to September 2016 and the American Community Survey 2012 to 2016 file.

^b^ High unemployment and poverty rate refer to zip codes in the top quartile of each state’s unemployment and poverty rate, respectively. Referrals to home health care is determined by the discharge disposition field on the Medicare Provider Analysis Review.

## Discussion

We found that only 54.0% of hospital discharges with a home health referral actually received home health care within 14 days of discharge. Among Black and Hispanic individuals and socioeconomically disadvantaged patients, the percentage who received home health was even lower. In addition, socioeconomically disadvantaged patients waited longer for their first home health visit. These results suggest that patients may face important differential barriers in access to home health care.

There are several potential explanations for low rates of successful referral to home health care. First, home health agencies may be unable or unwilling to serve all patients who are referred to them, potentially lacking staff or other resources needed. The lower referral rates among socioeconomically disadvantaged patients raise the possibility that home health agencies are selective about the patients for whom they provide care. There is a vast literature documenting that patients from racial/ethnic minority groups or socioeconomically disadvantaged backgrounds have worse access to care, even among fully insured patients.^13^ Home health agencies may view socioeconomically disadvantaged patients as overly complex to care for^14^ or may have a preference for serving higher socioeconomic status neighborhoods, resulting in lower rates of successful referral to home health among socioeconomically disadvantaged patients.

Second, our findings may be associated with patient preferences. Although patients may agree to home health care at the time of hospital discharge or accept a referral for other reasons, it is possible that once at home, they choose to forgo the care. Patients could believe that additional services are unnecessary or that home health services are low value, or they could prefer not to have health care practitioners in their home.^15^

Finally, the low rate of home health visits may be associated with data error. It is possible that incorrect data are being reported^16^ or patients are being referred who do not qualify for home health. However, some evidence suggests this is not the case. In our data, we find larger differences in receipt of home health care by patient characteristics than by hospital characteristics, which would not be expected if hospitals were systematically misreporting discharge referrals. Furthermore, the low visit rates and differences by patient characteristic persist when limiting the sample to a fee-for-service-enrolled population, for whom there are fewer constraints on coverage. In addition, patients’ discharge destination is more accurate for the institutional destinations in our data, such as skilled nursing facilities where 93.2% of patients referred to a skilled nursing facility at discharge receive care within 14 days, suggesting that the high discrepancy for home health care is not hospital reporting error. Even if one assumed that a similar 6.8% of patients are inaccurately coded as discharged to home health, this would suggest that only 60.8% of patients (rather than 54.0%) receive home health as intended.

Our findings are important for several reasons. Any benefit from receiving home health after hospital discharge is not accruing to a large proportion of people referred to home health care. The lower rates of home health visits among vulnerable patients is particularly troubling from an equity standpoint, where lower home health visits could contribute to disparities in health outcomes. These results are also troubling in that home health is increasingly being used as a substitute for institutional postacute care. Prior evidence suggests that patients discharged home with home health care rather than to a skilled nursing facility have higher readmission rates.^17^ The low rates of successful home health referral could result in even higher readmission rates as well as disparities in readmission rates.

### Limitations

This study has some limitations that should be discussed. We used the discharge destination code in MedPAR as a surrogate for referral to home health care. The data field indicates the practitioner team’s intent for postdischarge destination. The field is populated by the discharge planner, who also makes a referral to a home health agency, and is based on the medical team’s assessment of the need for home health care. Although some hospitals use a postdischarge reconciliation process, whereby the discharge destination code is reconciled with the actual discharge location, this is not uniform across hospitals. Thus, there may be some variation across hospitals in whether the field captures intent for postdischarge home health care or patients’ actual use of home health. This would underestimate the percentage of discharges that do not receive home health care when referred.

A related concern is that the discharge destination code may be inaccurate^18^ because of perverse incentives in Medicare’s Postacute Care Transfer Policy.^19^ However, this policy would tend to decrease the likelihood of hospitals coding the discharge destination as home health care, as discharge destinations that are coded as postacute care can decrease hospitals’ payments. This would exclude discharges that needed home health care from our sample and also result in an underestimate of the percentage of discharges receiving home health among those who need it. Thus, the discharge destination code provides a credible lower-bound estimate of the intent for home health care.

## Conclusions

In light of the coronavirus disease 2019 pandemic, experts have increasingly called for greater use of home health as a safer alternative to institutional postacute care.^20^ Furthermore, payment reforms continue to pressure hospitals to discharge patients home rather than to institutional settings. Thus, ensuring the availability of safe and equitable care will be crucial to maintaining high quality and safe care.
